# Attributional and attentional bias in children with conduct problems and callous-unemotional traits: a case–control study

**DOI:** 10.1186/s13034-020-00315-9

**Published:** 2020-03-10

**Authors:** Daniela Hartmann, Kathrin Ueno, Christina Schwenck

**Affiliations:** 1 Department of Special Needs Educational and Clinical Child and Adolescent Psychology, Justus-Liebig-University of Giessen, Otto-Behaghel-Straße 10 C, 35394 Giessen, Germany; 2 Department of Child and Adolescent Psychiatry, Psychosomatics, and Psychotherapy, University Hospital Frankfurt, Goethe-University, Frankfurt, Germany

**Keywords:** Conduct problems, Callous-unemotional traits, Conduct disorder, Oppositional defiant disorder, Social information processing

## Abstract

**Background:**

Children who are frequently aggressive or lack empathy show various deficits in their social information processing. Several findings suggest that children with conduct problems (CP) show a tendency to interpret ambiguous situations as hostile (hostile attribution bias) and have difficulties to disengage from negative stimuli (attentional bias). The role that additional callous-unemotional traits (CU-traits) play in these biases is yet unclear. Investigating both attentional and attributional aspects of social information processing in children can help us to understand where anomalies in the processing pathway occur and whether the biases are associated with CP and CU-traits separately or in an interactive manner.

**Methods:**

We compared three groups of children: (a) 25 children with CP and low levels of CU-traits (b) 25 children with CP and elevated levels of CU-traits (c) 50 gender (68% male), age (8–17 years) and intelligence score-matched typically developing children, on a pictorial emotional stroop task and a hostile attribution bias task.

**Results:**

In contrast to our predictions, there were no significant group differences regarding attentional biases or hostile attribution biases. Boys with CP and high levels of CU-traits showed a significantly higher hostile attribution bias compared to girls with CP and high levels of CU-traits. The attention bias to angry stimuli significantly correlated with the hostile attribution bias. Compared to the control group the CP group with low levels of CU-traits showed a significantly stronger association between the attention bias to angry stimuli and the hostile attribution bias.

**Conclusions:**

The current study provides evidence that boys with CP and high levels of CU-traits interpret ambiguous situations as more hostile than girls do. Our results further provide indications that the interaction of attentional and attributional biases in children with CP might contribute to their increased aggressive behavior.

## Background

Children with conduct disorder and oppositional defiant disorder exhibit various problem behaviors. According to the DSM-5 [1], the former is defined by symptoms including aggression towards animals and people, destructing the property of others, deceitfulness or theft, violations of rules and social norms and the latter by angry irritable mood, argumentative/defiant behavior, and vindictiveness. Children with such conduct problems (CP) form a highly heterogeneous group. For example, it is estimated that almost half of the individuals with CP exhibit high levels of callous-unemotional traits (CU-traits) [2]. CU-traits are related to the affective component of the concept of adult psychopathy and encompass characteristics, such as (a) a lack of empathy, (b) shallow affect, (c) a lack of remorse or guilt, (d) indifference towards one’s own performance [3].

Children with CP and high levels of CU-traits (CP–CU) and children with CP and low CU-traits (CP-only) have severe social deficits in common. However, children with CP–CU also differ from children with CP-only on certain aspects. They have a higher heritability of antisocial behavior [4–7], show reduced responsiveness to punishment [8, 9], as well as a reduced amygdala activity [10] and reduced startle response [11, 12] to distressing stimuli. These differences indicate that there are several distinct causal pathways, which can lead to the development of CP with and without CU-traits. Thus, understanding these differences is crucial in order to develop meaningful treatment strategies.

Many studies have looked at the social problems of children with CP and CU-traits in light of Dodge’s social information processing theory [13]. According to this theory, social information is processed in five steps: attention on and encoding of social cues, interpretation of social cues, response search, response evaluation, and enactment. The authors propose that a failure to regularly process either of these steps may lead to aggressive behavior. Even though, the literature has shown that children with CP show irregularities in all five steps [13–16], the vast majority of papers focuses on the encoding aspects of step one [17–20] and less so on the attentional aspects. However, there exists some evidence, which indicates that children with CP with and without CU-traits might, in fact, show differences in the attentional aspects of social information processing. For example, Hodsoll et al. [21] used an attentional capture task in which the children were presented with three pictures of faces, two female and one male, tilted to either the left or the right side. The faces depicted neutral or emotional (happy, afraid or sad) expressions. The task was to indicate to which side the target face (the male face) was tilted as quickly as possible. They showed that compared to TD children and children with CP-only, children with CP–CU seemed to be less distracted by task-irrelevant emotional facial expressions. Similarly, the interaction of high CU-traits and high aggression significantly predicted a reduced reaction time facilitation in a dot-probe task [22] and CU-traits were negatively correlated with facilitation effects in a lexical decision task [23]. Another paradigma, which was used to study attentional biases is the emotional stroop task. In this task, the participants are presented with neutral and aggression type words in different colors. The participants are instructed to ignore the content of the word and name the color as quickly as possible. The difficulty to disengage from a certain stimulus has been interpreted as an indication that these stimuli are more salient to the concerns of the individual as attentional resources seem to be automatically allocated towards these stimuli [24, 25]. As of yet, no study used the emotional stroop task to investigate attentional biases in children with CP. However, there exist studies looking at individuals with different levels of trait anger [24, 26, 27]. They found that individuals with high trait anger compared to individuals with low trait anger were significantly slower in naming the color of aggression type words relative to neutral words. Van Honk et al. [28] further showed that this attentional bias also holds if angry and neutral faces instead of words are presented.

Notably, children with CP might not only show attentional biases but also attributional biases. Thus, children with CP also differ in step two of the social information processing model. Previous studies have shown that aggressive children are more likely to interpret social situations with ambiguous provocative intent as hostile compared to non-aggressive children [29–32]. This tendency is referred to as the hostile attribution bias. A recent review by Martinelli et al. [14] found a more consistent relationship between reactive aggression and hostile attribution bias compared to proactive aggression and hostile attribution bias. As reactive aggression is commonly found in CP-only as well as CP–CU children [33] it can be hypothesized that children with CP-only and children with CP–CU both show an increased hostile attribution bias compared to TD children. However, studies, which directly investigated the hostile attribution bias in children with CP and CU-traits, produced inconsistent results. Frick et al. [31] reported an increased hostile attribution bias in boys with CP-only compared to all other groups, Cima et al. [32] observed an increased hostile attribution bias in CP–CU boys compared to CP-only boys, and Helseth et al. did not find any group differences [15]. As Cima et al. [32] only investigated boys, Frick [31] only observed group differences in the male portion of their study sample and Helseth et al. [15] did not observe any group differences in their mixed gender sample, gender might be a potential confounding factor and explain the discrepancies between these results.

Interestingly, little is known about the association between the hostile attribution bias and the attention bias for emotional cues. It seems intuitive to assume that a stronger attention bias towards hostile stimuli is correlated to more hostile interpretations of ambiguous situations. Additionally, considering that the attention towards a stimulus (step one) precedes the interpretation of a stimulus (step two) within the social information processing model [13], one could conclude that also the attention bias (step one) and the attribution bias (step two) depend on each other. In their integrative cognitive model, Wilkowski et al. [34] build on the social information processing model and propose that rather than a stepwise process it is an interaction of cognitive biases which leads to aggressive behavior. They suggest that an automatic hostile interpretation of ambiguous situations directly elicits anger but also leads to prolonged attention towards hostile stimuli, which again amplifies the level of anger and thus increases aggressive behavior. Thus, directly comparing the performance of children with CP and CP–CU on tasks involving attributional and attentional aspects of social information processing cannot only help us to uncover differences in two different steps of social information processing among children with CP with and without CU-traits but further provides us with the unique opportunity to investigate the association between attribution and attention biases. Therefore, in this study, we compared boys and girls with CP-only, CP–CU and typically developing children (TD) on two social information processing tasks. The pictorial emotional stroop task, similar to the task conducted by Van Honk et al. [28], focused on the attentional aspects of social information processing. With regards to the findings of an association between CU-traits and a missing attentional bias [21–23], we hypothesized that the performance of children with CP–CU, compared to children with CP-only or TD, is less affected by aggressive facial expressions. The objective of the second task was to expand our knowledge regarding the relationship between CP, CU-traits and the hostile attribution bias. Therefore we conducted a hostile attribution bias task similar to Frick et al. [31]. In line with the findings regarding the association between reactive aggression and the hostile attribution bias [14] and the findings that children with CP–CU as well as children with CP-only show reactive aggression [33], we hypothesized that children with CP-only as well as children with CP–CU interpret ambiguous situations significantly more often as hostile compared to TD children. In light of the integrative cognitive model [34], we further hypothesized that there is a positive correlation between the hostile attribution bias and attentional biases towards hostile stimuli. Since the association between the first two steps of the social information processing model has not been studied in children with CP and CU-traits, in an explorative manner we investigated, if the groups show differences in this association.

## Methods

### Subjects

A total of N = 181 participants were initially recruited for this study. The clinical participants were recruited from inpatient and outpatient clinics in Frankfurt am Main and Gießen (Germany), the children with typical development through advertisements in local newspapers and sports clubs. Exclusion criteria were any neurological or developmental disorder, an IQ below 80 or red-green color blindness. Prior to the study, all participants with CP had been diagnosed with either CD or ODD (according to DSM IV criteria) by experienced, independent clinicians. Children of the CP groups were matched with the TD group for age, gender, and IQ (case–control matching). Children were allocated to the CP–CU group if their CU traits were at least one SD above the mean of their gender and age group according to the german norms established by Ueno et al. [35]. In line with previous findings indicating more severe problem behaviors in children with CP and additional CU-traits compared to children with CP-only [36], the CP–CU group showed significantly higher externalizing behavior compared to the CP-only group. In order to exclude the possibility that group differences are due to differences in externalizing behavior rather than the level of CU-traits, we further matched the CP-only group and CP–CU group for the externalizing scale of the Child Behavior Checklist.

Fourteen participants did not meet inclusion criteria (IQ > 80) and 12 participants had to be excluded due to incomplete data resulting in a sample number of N = 155. After matching and group allocation the final sample consisted of N = 100 children (65% male) aged 8–17 years (M = 13.02; SD = 2.11) with n = 25 CP–CU, n = 25 CP-only and n = 50 TD children.

Four children with CP–CU and seven children of the CP-only group took medications on the day of the experiment (two children took antidepressants, three atypical psychotics, six methylphenidates, see Additional file 1: Table S1). 65% of the children with CP showed at least one comorbidity, ADHD (35%) being the most common one (All comorbidities of the CP-sample are presented in Additional file 2: Table S2). Prior to the experiment all participants and their parents gave written informed consent. The study was approved by the local ethics committee. Each participant received a small monetary compensation after completion of the experiment (Table 1).

**Table 1:** Participant descriptive

	CP–CU (N = 25)	CP-only (N = 25)	TD (N = 50)	F-value/χ^2^-value
Age	12.75 (2.02)	13.35 (2.33)	13.20 (2.01)	0.57
IQ	100.80 (10.90)	106.08 (13.86)	106.58 (13.36)	1.38
Verbal IQ	102.16 (11.85)	100.04 (12.88)^a^	107.60 (12.04)^b^	3.73*
Male (%)	72	64	68	0.368
ICU scale				
Raw score	35.36 (5.86)^a^	19.20 (7.15)^b^	14.46 (6.41)^c^	88.03***
T-score	68.95 (5.12)^a^	49.56 (8.73)^b^	43.06 (8.93)^c^	96.38***
CBCL ext				
Raw score	21.28 (8.57)^a^	16.60 (6.54)^b^	4.9 (3.99)^c^	70.68***
T-score	67.64 (6.36)^a^	64.20 (5.75)^a^	49.20 (7.69)^b^	74.28***
CBCL int				
Raw score	11.20 (6.97)^a^	10.04 (6.09)^a^	4.38 (4.12)^c^	16.63***
T-score	62.72 (7.77)^a^	60.60 (7.90)^a^	51.12 (8.46)^b^	21.29***

F-value is from one-way ANOVAs (*df*: 2, 107); χ^2^-value is from Chi square for categorical variables (gender). Different superscripts (a, b, c) denote significant group differences in post hoc pairwise comparisons

*ICU* Inventory of callous unemotional traits, *CBCL* Child Behavior Checklist (external and internal problem behavior)

* *p *< 0.05

*** *p* < 0.001

### Measures

#### Diagnostic interview for mental disorders in children and adolescents (Kinder-DIPS)

The Kinder-DIPS [37] is a semi-structured interview covering frequent mental disorders according to the DSM-IV and ICD-10 in children and adolescents aged 6–18 years. It has an overall satisfactory to very good interrater reliability (κ = 0.48–0.88, Yule’s-Y = 0.89–1.0) [37]. In the current study, the parent-version was applied to confirm the CD/ODD diagnosis in the clinical sample and to assess comorbid disorders. The parents of the TD children, only completed the interview questions of the Kinder-DIPS which are specific to the diagnosis of CD and ODD to rule out any CD/ODD diagnosis in the TD group.

#### Inventory of Callous and Unemotional Traits (ICU)

The ICU [38, 39] is currently the only validated questionnaire that assesses CU-traits in children and adolescents with parent-, teacher- and self-report versions being available in German. It consists of 24 items rated on a four-point Likert scale (0 = do not agree at all, 3 = strongly agree). The total score of the parent-version was used to perform group-allocation based on the German norms established by Ueno et al. [35]. For the total score of the German ICU the validity and the internal consistency were proven to be between acceptable (α = 0.77) [39] and good (α = 0.830) [35]. In our own sample, we obtained good internal consistency for the total ICU score (α = 0.879).

#### Child Behavior Checklist (CBCL/4-18, 1998)

The CBCL [40] consists of 113 items which parents rate on a three-point Likert scale (0 = do not agree, 2 = agree). It assesses problem-behavior in children aged 4–18 years and covers nine subscales as well as two higher-order subscales for internalizing and externalizing problem behavior. In the current study, the total T-score (T > 60) was used to rule out behavioral problems in the TD group. The CBCL has a satisfying validity and good reliability for the internalizing and externalizing problem behavior as well as the total score (> 0.80) [41]. We obtained good reliability for the externalizing (α = 0.858), acceptable values for the internalizing subscales (α = 0.795) and good values for the total score (α = 0.895).

#### Culture fair intelligence test (CFT-20R)

The CFT-20R [42, 43] assesses the general intelligence of children and adults from age 8.5 on. In the current study, the short version of the CFT-20R (part I), as well as the verbal test, was used. The short version consists of 56 items of non-verbal visual puzzles which are divided into four subtests. The CFT-20R shows a high internal consistency (> 0.80) and satisfying validity [43].

### Procedure

Prior to the experiment, all parents completed the CBCL and the ICU in a separate room. Parents of children with CP completed the Kinder-DIPS [37] and parents of the TD children completed the interview questions of the Kinder-DIPS which are specific to the diagnosis of CD and ODD. As a measure of verbal and nonverbal IQ, each participant completed the CFT-20R [43] before the computer experiment was started.

Each participant was positioned in a 90 cm distance from a computer screen (ASUS VH242H, 24’’, 1920 × 1080 Pixel, 60 Hz). The stimuli of both tasks were presented with Presentation^®^ software (Version 18.0, Neurobehavioral Systems, Inc., Berkeley, CA, https://www.neurobs.com).

### Pictorial emotional stroop task

**Fig. 1:**
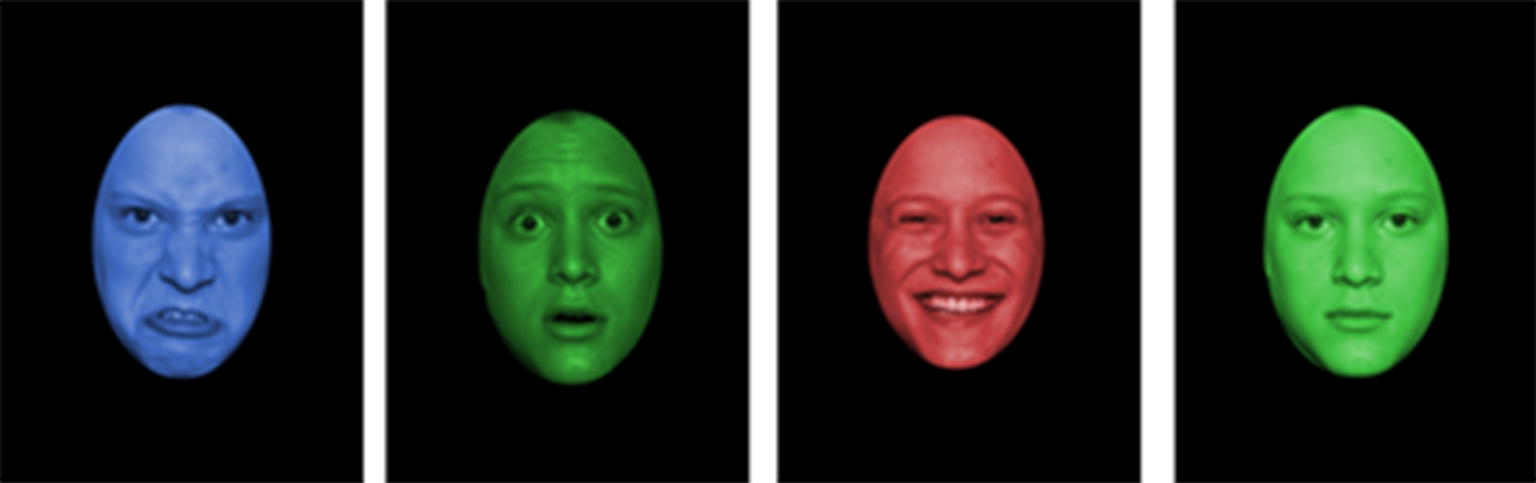
Stimuli of the pictorial emotional stroop task. Each facial expression (anger, fear, happiness, and neutral) was presented once in red, green, and blue (Pictures were chosen from the Karolinska Directed Emotional Faces database (Lundqvist et al. [44]) and adapted as described in “Methods” section)

To measure to what extent children are influenced by implicit social information processing we applied a pictorial emotional stroop task, similar to Van Honk et al. [28]. Thus, in contrast to the traditional emotional stroop task, we did not use emotional words but presented a single facial expression (angry, fearful, happy or neutral) in one of three different colors (blue, red, green) and asked the children to indicate the color of the stimulus as quickly as possible. Thus, the facial expressions served as the affective distractor and the color of the expressions as the target. The stimuli were chosen from the Karolinska Directed Emotional Faces database [44]. Four male and four female actors (2 × 4), each depicted angry, fearful, happy and neutral facial expressions. We cropped each picture into an oval shape to have only the face and no hair visible. We then produced three color versions of each picture (blue, red or green) resulting in a total of 96 picture-variants (2 × 4 × 4 × 3) (Fig. 1). Each stimulus was presented at the center of a black screen until the participant chose the correct color. The participants indicated the color of the stimulus by pressing one out of three buttons on a keyboard. After each correct response, a fixation cross was presented for 500 ms. If the participant chose the wrong color, the word “wrong” appeared underneath the stimulus and the stimulus remained on the screen until the correct color was chosen. The task consisted of three blocks of 32 trials each. In between the blocks, the children were able to take a break for any length of time. The implicit attentional capture task was preceded by a baseline block consisting of 25 trials to measure potential group-differences in the general reaction time. In the baseline block colored pictures depicting neutral objects (differently shaped bottles), instead of emotional faces, were shown.

In order to obtain a bias score for each of the three emotions, we first removed individual trials in which an incorrect response was given (7.8% of all trials) as well as correct trials with reaction times reflecting impulsiveness (< 150 ms) or inattentiveness (> 2000 ms) (0.9% of all trials). We then subtracted the mean reaction time to the neutral faces from the mean reaction time to the emotional faces.

### Hostile attribution bias task

To measure the hostile attribution bias, we used a modified version of the Intent Attribution Measure developed by Crick and Dodge [13]. The children were presented with ten stories of ambiguous situations. Two stories were derived from the study of Frick et al. [31], the other eight stories we adapted from the original stories in order to make them more age-appropriate (see Additional file 3: S3). The task thus consisted of a set of ten stories. The gender of the protagonist in the story always corresponded to the gender of the participant. After each story, the children were asked to answer three questions. In the first question, the children were asked about the intention of the protagonist’s action. They could choose among four answers—two implying a hostile intent and two implying a benign intent. All answers appeared in a randomized order. In the second question, the children were asked if they believed that the protagonist’s action was or was not intended to be mean. In the third question, the participants were requested to rate on a 3-point Likert scale how angry (not at all angry, a little angry, very angry) they would feel if they found themselves in the situation of the protagonist. This question was included as a measure of anger reactivity. The stories and questions were visually presented on a computer screen, answers to the questions were given using the keyboard.

We coded each benign answer to question one and two with zero and each hostile answer with one. The hostile attribution bias score was calculated by summing up the individual answers, resulting in a score between 0 and 10. Thus, more hostile answers to questions one and two resulted in a higher score indicating a stronger hostile attribution bias. The angry reactivity score was obtained accordingly by summing the answers to question three.

### Statistical analysis

All statistical analyses were performed using SPSS version 26 [45].

### Pictorial emotional stroop task

A 3 (group) × 3 (emotion) repeated measures analysis of variance (ANOVA) with group (CP–CU, CP-only, TD) as between-subjects variable and the bias score for each emotion (anger, fear, happiness) as within-subjects variable was conducted. As the test variables followed a multivariate normal distribution, observations were independent and Mauchly’s test for sphericity did not become significant, all assumptions for repeated measures ANOVA were met. Given previous findings that gender influences the processing of emotional faces [46], correlations were calculated between gender and bias scores. As gender did not significantly correlate with either of the bias scores, we did not account for gender in further analysis.

To determine if the bias scores of the participants showed a true interference (= a positive bias score with significantly slower reaction time to emotional stimuli compared to neutral stimuli) or true facilitation (= a negative bias score with significantly faster reaction time to emotional stimuli compared to neutral stimuli) an additional 3 (group) × 4 (stimulus type) repeated measures analysis of variance (ANOVA) with group (CP–CU, CP-only, TD) as between-subjects variable and the mean reaction times over stimulus type (anger, fear, happiness, neutral) as within-subjects variable was conducted.

### Hostile attribution bias task

To investigate if the groups significantly differed in their hostile attribution, conceptualized by the hostile attribution bias score, we conducted an ANOVA with the hostile attribution bias score as dependent and group as independent variable. Given previous findings that group differences for the hostile attribution bias might only be observed in boys and not in girls [31], and as gender and the hostile attribution bias significantly correlated (*r* = − 0.178, *p* = 0.038), we included gender as a second independent variable.

#### Association of hostile attribution bias and attentional bias

In order to investigate the association of the hostile attribution bias and the attentional bias towards angry stimuli, we calculated bivariate Pearson correlations between the hostile attribution bias score and the attribution bias score for angry stimuli. We also calculated correlations between the angry reactivity score (question three) and the attentional bias towards angry stimuli. To explore whether we might be able to observe differences among the groups regarding the association of the biases, we further calculated Pearson correlations for each group separately and compared them using Fisher’s z-test [47].

## Results

### Pictorial emotional stroop task

The comparisons between the mean reaction time to the neutral and the three emotional face types revealed no significant effect of group on emotion. The repeated measures ANOVA with the bias scores as within-subject variable revealed a significant main effect of emotion *F* (2194) = 5.564, *p* = 0.004, *partial* η^2^ = 0.054, but not group *F* (2, 97) = 2.077, *p* = 0.131, *partial* η^2^ = 0.041. There was no significant interaction of emotion × group *F* (4, 194) = 0.028, *p* = 0.998, *partial* η^2^ = 0.001. Bonferroni adjusted pairwise comparisons showed that the participants had significantly lower bias scores when viewing happy emotional faces (*M *= 18.34, *SD *= 5.07) compared to fearful (*M *= 36.70, *SD *= 6.12) *p* = 0.020 and angry emotional faces (*M *= 38.11, *SD *= 5.86) *p* = 0.004. The error rates were generally very low (on average 89 out of 96 trials correct) and the groups differed neither in their total error rate nor in the error rates for the individual emotions. The additional repeated measures ANOVA with the mean reaction times as within-subject factors revealed a significant main effect of stimulus type *F* (3291) = 16.902, *p* < 0.000, *partial* η^2^ = 0.148. Subsequent Bonferroni adjusted pairwise comparisons showed that all reaction times to emotional stimuli were significantly slower (p < 0.01) compared to neutral stimuli (see Table 2 for mean reaction times). Thus, the bias scores of the participants show a true interference effect for all three emotions.

**Table 2:** Mean reaction times and standard deviations for all emotions and groups

	RT		
	CP–CU	CP-only	TD
Anger	634.00 (133.23)	616.17 (144.46)	637.21 (140.17)
Fear	630.59 (142.49)	615.74 (128.71)	636.82 (143.59)
Happy	615.48 (123.55)	595.28 (114.68)	617.32 (124.93)
Neutral	595.67 (119.12)	568.11 (96.50)	609.26 (134.33)

*RT* reaction time in milliseconds

### Hostile attribution bias task

**Fig. 2:**
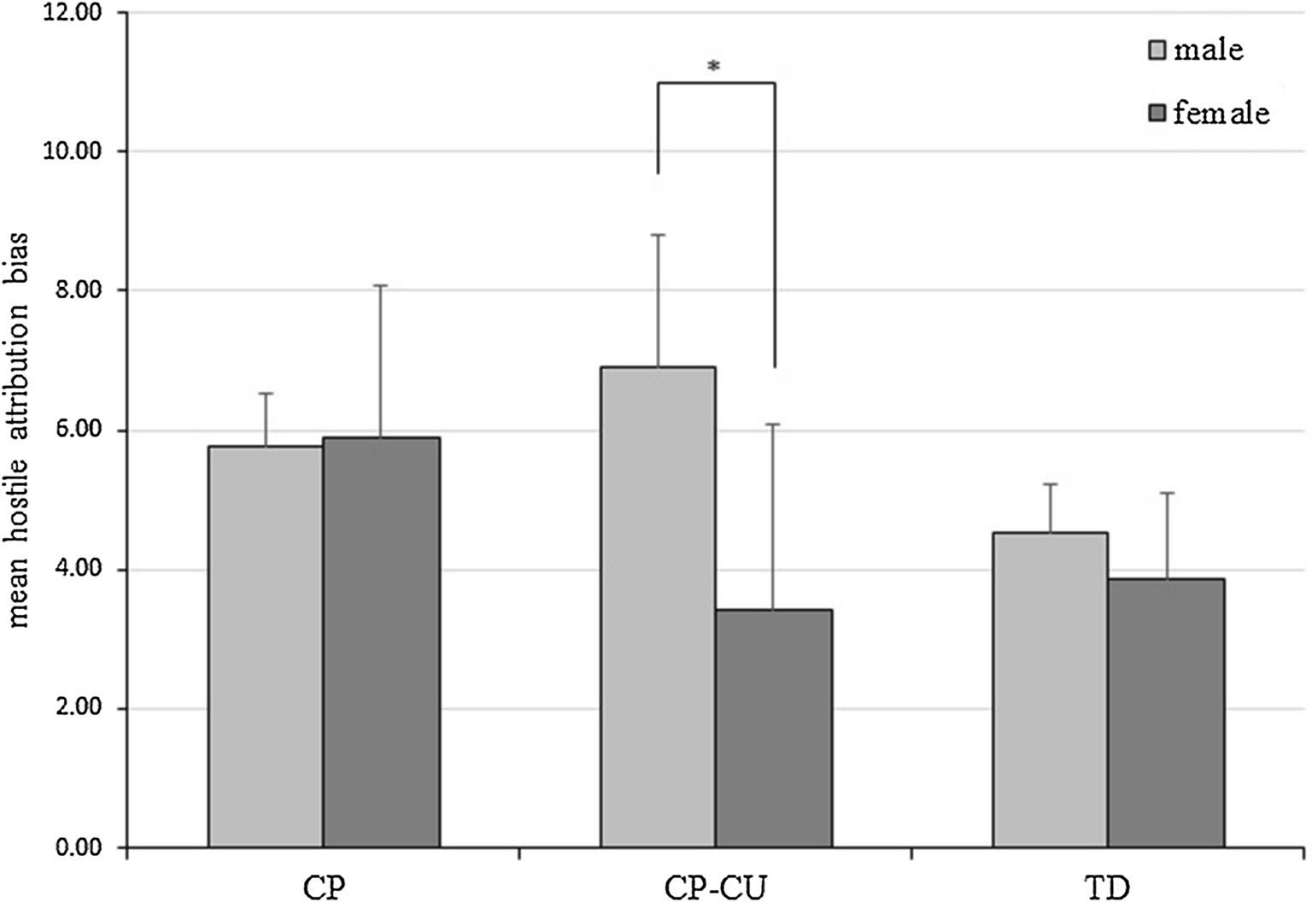
Mean hostile attribution bias scores. Comparison of mean hostile attribution bias scores between male and female participants of each group. *p < 0.05

The ANOVA revealed a significant effect for gender *F* (1, 94) = 4.552, *p *= .035 *partial* η^2^ = 0.046 and a marginally significant effect for group *F* (2, 94) = 2.759, *p* = 0.066, *partial* η^2^ = 0.056. The interaction of group and gender did not reach significance *F* (2, 94) = 2.610, *p* = 0.079, *partial* η^2^ = 0.053. Post-hoc tests with Bonferroni correction revealed that the hostile attribution bias scores of the CP–CU group (*M *= 5.92, *SD *= 4.17) and CP-only group (*M *= 5.80, *SD *= 2.55) did not significantly differ from each other (*p* = 1.000). Neither did the comparison between CP-only and TD group (*M* = 5.52, SD = 2.32; *p *= 0.089). However, the CP–CU group showed a trend for a higher hostile attribution bias compared to the TD group (*p *= 0.057). In order to investigate the significant gender effect within each group we conducted multiple independent t-tests. Within the CP–CU group, boys (*M* = 6.89, *SD* = 4.09) showed a significantly higher hostile attribution bias compared to girls (*M* = 3.43, *SD* = 3.51), t (23) = 1.970, *p* = 0.030 (Fig. 2).

### Association of hostile attribution bias and attentional bias

There was a significant relationship between the hostile attribution bias and the attention bias towards angry stimuli, r = 0.275, *p* = 0.003, as well as between the angry reactivity score and the attention bias towards angry stimuli, r = 0.204, *p* = 0.021. There was also a small correlation between the hostile attribution bias and the attention bias towards happy stimuli r = 0.170, *p* = 0.045. When analyzing the groups separately, only the correlation between the hostile attribution bias and the attention bias towards angry stimuli within the CP-only group became significant, r = 0.545, *p* = 0.002. According to Fisher’s z-test the association between the hostile attribution bias and the attention bias towards angry stimuli was significantly stronger for the CP-only group compared to the TD-group (r = 0.067) with p = 0.018, but not compared to the CP–CU group (r = 0.247).

## Discussion

The purpose of the current study was to examine attributional and attentional biases in social information processing via a pictorial emotional stroop task and a hostile attribution bias task in children with CP–CU, CP-only, and TD. We hypothesized that children with CP-only would show a significantly higher attentional bias compared to the control group. We further expected that children with CP–CU would show significantly less attentional interference compared to the TD group and CP-only group.

In contrast to our expectations, the groups did not significantly differ concerning the attentional bias. Instead, we were able to find a significant attentional bias to negative facial expressions for all participants. Thus, irrespective of the group, negative emotional faces interfered with children’s performance on the actual task. Our findings are not in line with previous findings indicating that children with CP–CU are less affected by attentional biases to emotional stimuli [21–23]. In comparison to Hodsoll et al. [21], our CP–CU sample consisted of children with overall lower levels of CU-traits (*M* = 35.36, *SD* = 5.86 vs. *M *= 49.5, *SD* = 7.7). This might indicate that differences in the attentional bias are only observable in children with very high levels of CU-traits. However, it has to be considered that Hodsoll et al. used the teacher version of the ICU, which generally produces higher scores compared to the here applied parent version [35]. Furthermore, Kimonis et al. [22] showed that the interaction of CU-traits and aggression negatively predicts the facilitation to distressing cues in a dot-probe task in a sample of detained individuals with only moderate levels of self-reported CU-traits (*M *= 23.23, *SD* = 7.85). Interestingly, Loney et al. [23], only reported indications of an association between a lower attentional bias and CU-traits if they controlled for impulsivity. However, reanalyzing our attentional bias data with impulsivity as covariate, did not change our results in a meaningful way.

Furthermore, our findings are also not compatible with the findings of previous studies, which conducted an emotional stroop task with adults with high and low levels of anger and observed a stronger interference for high anger individuals [24, 28]. In comparison to these previous studies, we did not differentiate between groups according to the level of trait anger, but based on a clinical diagnosis of CP. This could indicate that only individuals with high levels of trait anger but not individuals with high levels of behavioral aggression show increased difficulties to disengage from negative emotional stimuli. However, this assumption needs to be considered with caution. Even though angry irritable mood is a core symptom of oppositional defiant disorder and trait anger is highly predictive of aggressive behavior [48, 49], we did not assess trait anger in our sample. Future studies should investigate attentional biases including measures of state and trait anger as well as aggressive behavior in order to determine potential differences.

Regarding our second task, we expected both CP groups to show a higher hostile attribution bias compared to the TD group. Although the overall group effect became only marginally significant, we observed a significantly higher hostile attribution bias in the CP–CU group compared to the TD group. The comparison between the CP-only group and TD group almost approached significance. As both children with CP–CU as well as children with CP-only show increased levels of reactive aggression [33] our findings of a tendency for a higher hostile attribution bias in both of these groups fit the results of a meta-analysis, showing that reactive aggression is associated with higher levels of the hostile attribution bias [14]. Nonetheless, our findings contradict the previous finding of a stronger hostile attribution bias in CP-only compared to CP–CU children [31], the findings of a higher hostile attribution bias in CP–CU compared to CP-only children [32] and the findings of no group differences at all [15]. Our study overcame several shortcomings of these previous studies. Due to their small sample size, Helseth et al. [15] were not able to consider potential gender effects. Cima et al. [32] similarly failed to consider possible gender confounds as their study sample consisted of exclusively male participants. In line with Frick et al. [31], we observed an association between gender and the hostile attribution bias, with boys of the CP–CU group showing a significantly higher hostile attribution bias compared to girls. Interestingly, we only observed this gender difference within the CP–CU group but not the CP-only group. These results could indicate that children with CP–CU might benefit from gender-specific interventions.

As expected, our analysis revealed that a higher attention bias towards angry stimuli is associated with a higher hostile attribution bias as well as a higher anger reactivity score. Thus, children who interpreted ambiguous situations as hostile more often also showed higher interference when presented with angry facial expressions. We do not have a ready explanation of the correlation between attentional biases to happy stimuli and the hostile attribution bias. However, this correlation was rather small and only was significant when investigating the whole sample and not in the individual groups. Notably, a significant association between the hostile attribution bias and the attention bias could only be observed for anger stimuli in the CP-only group. Furthermore, this association was significantly stronger within the CP-only group compared to the TD group. Thus, it seems that even though the children with CP-only did not show a higher attentional bias or a higher hostile attribution bias compared to the TD participants, this group of children has an especially strong connection between attributional and attentional biases. This could indicate, that the interaction of these biases is what leads to the aggressive behavior in children with CP-only, as the integrative cognitive model [34] proposes. As children with CP–CU did not show a significant correlation between the attribution bias and attention bias and did not significantly differ in their association compared to the TD group, it is likely that their aggressive behavior results from a different processing pathway. However, due to the explorative manner of this investigation and the fact that the strength of the association between the CP-only and the CP–CU group did not significantly differ, this interpretation needs to be considered with caution.

### Strengths and limitations

The current study is, to our knowledge, the first study to compare children with CP with and without CU-traits on social information processing tasks involving two different cognitive biases. Investigating both attentional and attributional aspects of social information processing can help us in precisely identifying where anomalies in the processing pathway occur and thus provide us with valuable information to guide the development of successful treatments. In contrast to most previous studies, we investigated a mixed study sample and thus took the possible influence of gender into account. Aside from the previously mentioned limitations, the following considerations should be noted. According to post hoc power analysis using G*power [50], our study sample might be too small to detect significant group differences. As we could only rely on one study to estimate the effect size for the emotional stroop task [28] we used the reported effect size (f = 0.37) as well as a more conservative estimation (f = 0.25). Similarly, we used a more liberal (f = 0.35) and a more conservative estimation (f = 0.25) for the hostile attribution bias task, as a meta-analysis of the hostile attribution bias [51] indicated that effect sizes across studies are highly variable and depend on various factors. Thus, with an alpha level of 0.05, our sample size achieved a power between 0.67 and 0.91 to detect group differences in the emotional stroop task and a power between 0.59 and 0.88 to detect group differences in the hostile attribution bias task.

We did not counterbalance the order of the tasks, as we did not expect the tasks to influence each other. All children first performed the emotional stroop task and then the hostile attribution bias task. This order was chosen as the hostile attribution bias task does not require fast reactions and is thus less affected by a possible drop in concentration.

Similarly to most studies including children with CP, we cannot completely rule out that motivational factors may have influenced the results. However, the CP-only, as well as CP–CU children did not differ from the TD children in their general reaction time, in the number of mistakes in the pictorial emotional stroop task and they did not show any answer-patterns in the hostile attribution bias task that would indicate that e.g. they simply pressed the same button to answer each question. We did not assess reactive aggression within our sample, thus it is possible that the CP–CU group and CP-only group slightly differed on their level of reactive aggression. A slightly lower level of reactive aggression in the children with CP-only, for example, could explain why the difference to the TD group in the hostile attribution bias task did not become significant. As we were primarily interested in the investigation of subgroups of children with CP, we did not include a CU-only group in our study. However, future studies should consider including a CU-only group when investigating social information processing in order to assess in how far CU-traits influence social information processing in the absence of CP.

## Conclusions

Even though we did not observe any significant group differences regarding attentional and attributional biases, our results provide first indications that attentional and attributional biases are linked and that the strength of this association might be especially strong in children with CP-only. Furthermore, the current study provides evidence that there is a significant gender effect for the hostile attribution bias with boys with CP–CU showing a significantly higher hostile attribution bias compared to girls with CP–CU. This finding could further implicate that in contrast to girls, boys with CP–CU might benefit from treatment programs that aim at modifying the child’s way of interpreting ambiguous situations. Future studies should include girls in their study sample and consider comparing subgroups of children with CP on multiple measures of cognitive biases. Such investigations can help us understand how processing biases interact and in how far differences in these interactions might represent distinct pathways to aggressive behavior.

## Supplementary information

**Additional file 1.** Number of participants currently taking medication.

**Additional file 2.** Number of comorbidities according to the Kinder-DIPS.

**Additional file 3.** Hostile attribution bias stories.

## Data Availability

The datasets used and/or analyzed during the current study are available from the corresponding author on reasonable request.
