# Supplementary material for: Attributional and attentional bias in children with conduct problems and callous-unemotional traits: a case–control study
Source: Child Adolesc Psychiatry Ment Health. 2020 Mar 10;14:9. doi: 10.1186/s13034-020-00315-9 (PMC7063755; doi:10.1186/s13034-020-00315-9)
Supplement: Supplementary file 1 — Additional file 1. Number of participants currently taking medication. [file 13034_2020_315_MOESM1_ESM.docx]

**Attributional and attentional bias in children with conduct problems and callous-unemotional traits: a case-control study.**

Daniela Hartmann^1^, Kathrin Ueno^2^, Christina Schwenck^1,2^

^1^ Justus-Liebig-University of Giessen, Department of Special Needs Educational and Clinical Child and Adolescent Psychology

² Department of Child and Adolescent Psychiatry, Psychosomatics, and Psychotherapy, University Hospital Frankfurt, Goethe-University, Frankfurt am Main, Germany

Corresponding author:

[Daniela.Hartmann@psychol.uni-giessen.de](mailto:Daniela.Hartmann@psychol.uni-giessen.de)

**Supporting information**

Table S1: *Number of participants currently taking medication.*

|  | CP-CU (N=25) | CP-only (N=25) | TD (N=50) |
| --- | --- | --- | --- |
| Phenylethylamines (methylphenidate) | 3 | 3 | 0 |
| Atypical neuroleptics | 1 | 2 | 0 |
| SSRIs | 0 | 2 | 0 |
