# Supplementary material for: Attributional and attentional bias in children with conduct problems and callous-unemotional traits: a case–control study
Source: Child Adolesc Psychiatry Ment Health. 2020 Mar 10;14:9. doi: 10.1186/s13034-020-00315-9 (PMC7063755; doi:10.1186/s13034-020-00315-9)
Supplement: Supplementary file 2 — Additional file 2. Number of comorbidities according to the Kinder-DIPS. [file 13034_2020_315_MOESM2_ESM.docx]

**Attributional and attentional bias in children with conduct problems and callous-unemotional traits: a case-control study.**

Daniela Hartmann^1^, Kathrin Ueno^2^, Christina Schwenck^1,2^

^1^ Justus-Liebig-University of Giessen, Department of Special Needs Educational and Clinical Child and Adolescent Psychology

² Department of Child and Adolescent Psychiatry, Psychosomatics, and Psychotherapy, University Hospital Frankfurt, Goethe-University, Frankfurt am Main, Germany

Corresponding author:

[Daniela.Hartmann@psychol.uni-giessen.de](mailto:Daniela.Hartmann@psychol.uni-giessen.de)

**Supporting information**

Table S2: *Number of comorbidities according to the Kinder-DIPS.*

| Comorbidities within the CP group | CP-CU | CP-only |
| --- | --- | --- |
| Attention deficit/hyperactivity disorder | 11 | 8 |
| Specific phobia | 4 | 3 |
| Enuresis | 3 | 1 |
| Generalized social phobia | 3 | 0 |
| Major depression | 2 | 1 |
| Selective mutism | 1 | 1 |
| Transient tic disorder | 1 | 1 |
| Dysthymia | 2 | 0 |
| Anxiety disorder | 2 | 0 |
| Insomnia | 0 | 1 |
| Separation anxiety | 1 | 0 |
| Obsessive Compulsive disorder | 0 | 1 |
| Encopresis | 1 | 0 |
