# Supplementary material for: Attributional and attentional bias in children with conduct problems and callous-unemotional traits: a case–control study
Source: Child Adolesc Psychiatry Ment Health. 2020 Mar 10;14:9. doi: 10.1186/s13034-020-00315-9 (PMC7063755; doi:10.1186/s13034-020-00315-9)
Supplement: Supplementary file 3 — Additional file 3. Hostile attribution bias stories. [file 13034_2020_315_MOESM3_ESM.docx]

**Attributional and attentional bias in children with conduct problems and callous-unemotional traits: a case-control study.**

Daniela Hartmann^1^, Kathrin Ueno^2^, Christina Schwenck^1,2^

^1^ Justus-Liebig-University of Giessen, Department of Special Needs Educational and Clinical Child and Adolescent Psychology

² Department of Child and Adolescent Psychiatry, Psychosomatics, and Psychotherapy, University Hospital Frankfurt, Goethe-University, Frankfurt am Main, Germany

Corresponding author:

[Daniela.Hartmann@psychol.uni-giessen.de](mailto:Daniela.Hartmann@psychol.uni-giessen.de)

**Supporting information**

S3: *Hostile attribution bias stories.*

Story 1

One of your classmates is having a big party, a lot of people from your class are invited to. You haven’t received an invitation yet.

Question 1: Which of the following reasons might be possible for your classmate to have?

1. He forgot to send you an invitation
2. He didn’t want you at the party.
3. He only was allowed to invite a certain amount of people.
4. He wanted you to feel excluded.

Question 2: Do you think your classmate’s behavior is meant to be …

1. evil
2. not evil

Question 3: How angry would you be, if that situation really happened to you?

1. not at all
2. a little bit
3. a lot

Story 2

During the lesson, you are looking out of the window and the teacher who often scolds you tells you to pay attention.

Question 1: Which of the following reasons might be possible for your teacher to have?

1. He is going after you and uses every opportunity to scold you.
2. He thinks you won’t understand the lesson like that.
3. He wants you to pay attention to get better grades.
4. He wants to make a fool of you in front of the others.

Story 3

You brought your new MP3-Player to school and during the break, you leave it at your table. After the break one of your classmates tells you, that he pushed it off the table. It is broken now.

Question 1: Which of the following reasons might be possible for your classmates to have?

1. He accidentally pushed the MP3-Player off the table, because someone else pushed him against the table.
2. He was jealous and broke it intentionally.
3. He dropped it while having a closer look at the MP3-Player.
4. He doesn’t like you.

Story 4

Your friend is telling you that he went to an amusement park with a lot of people from your class on the weekend. You have been wanting to go there for a long time and told him this not too long ago.

Question 1: Which of the following reasons might be possible for your friend to have?

1. He didn’t want you to come with them, because he wanted you to get jealous.
2. He thought, that you already had plans for the weekend.
3. He wanted you to feel excluded.
4. He forgot, that you wanted to go to this amusement park.

Story 5

Your friend got a new bike for his birthday and you are looking at him, while he is trying it out. After he rode it for a while, you want to ride it as well but he won’t let you.

Question 1: Which of the following reasons might be possible for your friend to have?

1. He is very careful because he is worried about his new bike.
2. He doesn’t want you to have fun with his new bike as well.
3. He wants you to get angry.
4. His parents told him to not let anybody else ride his new bike.

Story 6

You ask your parents whether you can have a new gaming console, which you’ve been wishing for, for a long time. They tell you “No”.

Question 1: Which of the following reasons might be possible for your parents to have?

1. They don’t take your wishes seriously.
2. They think it’s not good for you to have a gaming console and play a lot.
3. They don’t care, that the gaming console would mean a lot of fun for you.
4. They want you to do other things in your free time.

Story 7

While in school you want to write something down and realize, that you forgot your writing pad. You ask your desk neighbor whether you can have a sheet of paper, but he doesn’t want to give you one.

Question 1: Which of the following reasons might be possible for your desk neighbor to have?

1. He wants you to get in trouble because you can’t write anything down.
2. He only has a few sheets left.
3. He wants you to make a fool of yourself because you have to ask loudly in class now.
4. He is concentered while writing something down and doesn’t have time to give you a sheet.

Story 8

While standing on the schoolyard you open your bottle and spill some of your drink on your pants. At first, the others don’t notice it but one of your friends laughs out loud. Now everybody else is aware of it as well.

Question 1: Which of the following reasons might be possible for your friend to have?

1. He wanted to tease you.
2. He thought that it was funny, but didn’t want the others to get aware of it.
3. He thought you wouldn’t take it too seriously if he laughed.
4. He wanted the others to be aware of your little accident and to laugh at you as well.

Story 9

Your parents want you to help with the housework on a Saturday morning, but you tell them that you would rather watch TV. Your parents won’t let you and tell you, that you can watch TV after the housework is done.

Question 1: Which of the following reasons might be possible for your parents to have?

1. They don’t want you to watch too much TV, because they think that it’s not good for you.
2. They don’t want you to have fun on your free day.
3. They want you to help with the housework because they want you to learn how to take responsibility.
4. They want you to help with the housework so that they don’t have to do all of it.

Story 10

You lend a computer game to a friend and he gives it back to you. Now you want to play it at home, but recognize that the CD doesn’t work anymore because it has a scratch.

Question 1: Which of the following reasons might be possible for your friend to have?

1. He didn’t recognize, that the CD had a scratch and didn’t tell you because of that.
2. He intentionally scratched the CD.
3. He recognized the scratch but thought it was awkward to tell you.
4. He didn’t tell you, because he wanted to claim, that you destroyed it yourself.
